# Imaging Response to Contemporary Immuno-oncology Combination Therapies in Patients With Metastatic Renal Cell Carcinoma

**DOI:** 10.1001/jamanetworkopen.2022.16379

**Published:** 2022-06-10

**Authors:** Vishal Navani, Matthew Ernst, J. Connor Wells, Takeshi Yuasa, Kosuke Takemura, Frede Donskov, Naveen S. Basappa, Andrew Schmidt, Sumanta K. Pal, Luis Meza, Lori A. Wood, D. Scott Ernst, Bernadett Szabados, Thomas Powles, Rana R. McKay, Andrew Weickhardt, Cristina Suarez, Anil Kapoor, Jae Lyun Lee, Toni K. Choueiri, Daniel Y. C. Heng

**Affiliations:** 1 Tom Baker Cancer Centre, Department of Medical Oncology, University of Calgary, Calgary, Canada; 2 BC Cancer Agency, Vancouver, Canada; 3 Cancer Institute Hospital, Japanese Foundation for Cancer Research, Tokyo, Japan; 4 Department of Oncology, Aarhus University Hospital, Aarhus, Denmark; 5 Cross Cancer Institute, Department of Medical Oncology, University of Alberta, Edmonton, Canada; 6 Dana Farber Cancer Institute, Boston, Massachusetts; 7 City of Hope Comprehensive Cancer Center, Duarte, California; 8 Queen Elizabeth II Health Sciences Centre, Halifax, Canada; 9 London Regional Cancer Centre, London, Canada; 10 Barts Cancer Institute, Queen Mary University of London, London, United Kingdom; 11 Moores Cancer Center, University of California, San Diego, La Jolla; 12 Olivia Newton-John Cancer and Wellness Centre, Melbourne, Australia; 13 Vall d’Hebron Institute of Oncology, Universitat Autònoma de Barcelona, Barcelona, Spain; 14 Juravinski Cancer Centre, McMaster University, Hamilton, Canada; 15 University of Ulsan College of Medicine, Asan Medical Center, Seoul, Republic of Korea

## Abstract

**Importance:**

The association between treatment with first-line immuno-oncology (IO) combination therapies and physician-assessed objective imaging response among patients with metastatic renal cell carcinoma (mRCC) remains uncharacterized.

**Objective:**

To compare the likelihood of objective imaging response (ie, complete or partial response) to first-line IO combination ipilimumab-nivolumab (IOIO) therapy vs approved IO with vascular endothelial growth factor inhibitor (IOVE) combination therapies among patients with mRCC.

**Design, Setting, and Participants:**

This multicenter international cohort study was nested in routine clinical practice. A data set from the International Metastatic Renal Cell Carcinoma Database Consortium (IMDC) was used to identify consecutive patients with mRCC who received treatment with IO combination therapies between May 30, 2013, and September 9, 2021. A total of 899 patients with a histologically confirmed diagnosis of mRCC who received treatment with a first-line IOVE or IOIO regimen and had evaluable responses were included.

**Exposures:**

Best overall response to first-line IO combination therapy based on Response Evaluation Criteria in Solid Tumors, version 1.1.

**Main Outcomes and Measures:**

The primary outcome was the difference in treating physician–assessed objective imaging response based on the type of first-line IO combination therapy received. Secondary outcomes included the identification of baseline characteristics positively associated with objective imaging response and the association of objective imaging response with overall survival.

**Results:**

Among 1085 patients with mRCC who received first-line IO combination therapies, 899 patients (median age, 62.8 years [IQR, 55.9-69.2 years]; 666 male [74.2%]) had evaluable responses. A total of 794 patients had information available on IMDC risk classification; of those, 127 patients (16.0%) had favorable risk, 442 (55.7%) had intermediate risk, and 225 (28.3%) had poor risk. With regard to best overall response among all participants, 37 patients (4.1%) had complete response, 344 (38.3%) had partial response, 315 (35.0%) had stable disease, and 203 (22.6%) had progressive disease. Corresponding median overall survival was not estimable (95% CI, 53.3 months to not estimable) among patients with complete response, 55.9 months (95% CI, 44.1 months to not estimable) among patients with partial response, 48.1 months (95% CI, 33.4 months to not estimable) among patients with stable disease, and 13.0 months (95% CI, 8.4-18.1 months) among patients with progressive disease (log rank *P* < .001). Treatment with IOVE therapy was found to be independently associated with an increased likelihood of obtaining response (OR, 1.89; 95% CI, 1.26-2.81; *P* = .002) compared with IOIO therapy. The presence of lung metastases (odds ratio [OR], 1.49; 95% CI, 1.01-2.20), receipt of cytoreductive nephrectomy (OR, 1.59; 95% CI, 1.04-2.43), and favorable IMDC risk (OR, 1.93; 95% CI, 1.10-3.39) were independently associated with an increased likelihood of response.

**Conclusions and Relevance:**

In this study, treatment with IOVE therapy was associated with significantly increased odds of objective imaging response compared with IOIO therapy. The presence of lung metastases, receipt of cytoreductive nephrectomy, and favorable IMDC risk were associated with increased odds of experiencing objective imaging response. These findings may help inform treatment selection, especially in clinical contexts associated with high-volume multisite metastatic disease, in which obtaining objective imaging response is important.

## Key Points

**Question:**

Do contemporary first-line therapy options for metastatic renal cell carcinoma (mRCC) have different likelihoods of objective imaging response (complete or partial response), and is objective imaging response associated with overall survival?

**Findings:**

In this cohort study involving 899 patients with mRCC, treatment with immune checkpoint blockade plus vascular endothelial growth factor receptor inhibitor combination therapies was more likely to be associated with objective imaging response than doublet immune checkpoint blockade therapy after adjustment for baseline demographic and clinical characteristics, including International Metastatic Renal Cell Carcinoma Database Consortium risk criteria. Objective imaging response was associated with improvement in overall survival among patients receiving both types of therapy.

**Meaning:**

This study’s findings suggest that combination therapies with first-line immune checkpoint blockade plus vascular endothelial growth factor receptor inhibitor were more likely to be associated with objective imaging response than doublet immune checkpoint blockade therapy.

## Teaser

This cohort study uses data from the International Metastatic Renal Cell Carcinoma Database Consortium to assess the likelihood of objective imaging response to first-line immuno-oncology therapies among patients with metastatic renal cell carcinoma.

## Introduction

Improvements in overall survival (OS) among patients with metastatic renal cell carcinoma (mRCC) have been achieved by combination therapy approaches using agents targeting immune checkpoint blockade (ICB) and the vascular endothelial growth factor (VEGF) receptor.^1^ Patients and physicians have numerous first-line immuno-oncology (IO) treatment options, including doublet ICB therapy with ipilimumab-nivolumab (IOIO) or ICB therapy with VEGF receptor inhibitor (IOVE; including axitinib-avelumab, axitinib-pembrolizumab, cabozantinib-nivolumab, and lenvatinib-pembrolizumab) combination therapies. However, most patients eventually develop resistance to these therapies.^2^

Despite the challenges of using Response Evaluation Criteria in Solid Tumors (RECIST), version 1.1,^3^ to assess response to ICB therapy,^4,5^ RECIST was important in the clinical development of IOIO and IOVE therapies and is still used to define surrogate end points across clinical development in treatment approaches for mRCC.^6^ Although data exist to support a significant association between achievement of objective imaging response (ie, complete or partial response on imaging) and long-term survival among patients with mRCC receiving ICB therapy,^7^ this association has not been examined among a routine-practice population receiving treatment with first-line combination therapies.

Despite the meaningful improvements in OS that have been achieved by first-line combination therapies, up to 20% of patients have progressive disease as their best overall response, with clinical trial data suggesting that the all-cause mortality rate at 1 year is as high as 20%.^8^ In this context, biomarkers to predict either objective imaging response^9^ or survival benefit^10^ associated with first-line therapies are lacking, hampering attempts to improve the survival curve. Clinically accessible baseline characteristics associated with response may help tailor treatment selection. Any clinically meaningful difference in the likelihood of objective imaging response based on the type of first-line therapy received may inform therapeutic selection, especially if tumor reduction is necessary to prevent life-limiting disease progression and palliate tumor-induced symptoms. To address this evidence gap, we used the International Metastatic Renal Cell Carcinoma Database Consortium (IMDC) data set to compare the likelihood of obtaining objective imaging response to first-line IOIO vs IOVE therapies and identify baseline variables associated with physician-assessed objective imaging response.

## Methods

### Design and Patient Population

The IMDC is a large consecutive-patient observational cohort study conducted by treating physician investigators. The study has enrolled patients across multiple centers globally and requires all patients to have received a systemic therapy for mRCC. Data for the current cohort study were collected from May 30, 2013, to September 9, 2021, with statistical analysis performed in February 2022. The study was approved by the institutional review boards of each IMDC participating site. The Health Research Ethics Board of Alberta provided ethics approval for the IMDC sites in Alberta, Canada. All participants provided written informed consent. This study followed the Strengthening the Reporting of Observational Studies in Epidemiology (STROBE) reporting guideline for cohort studies.

Inclusion criteria for this study comprised a histologically confirmed diagnosis of mRCC and receipt of treatment with an approved first-line IOVE or IOIO regimen. All included patients were required to have evaluable imaging response, which was defined as the receipt of baseline imaging and at least 1 set of imaging studies after initiation of systemic therapy. Patients without data regarding treatment duration were excluded from the survival analysis.

### Outcome Measures

The primary outcome was the difference in physician-assessed objective imaging response based on the type of first-line IO combination therapy received. Secondary outcomes included the association of objective imaging response with OS and time to next treatment (TTNT) and baseline characteristics associated with objective imaging response.

Version 1.1 of RECIST^3^ was the imaging assessment framework used to define response to first-line therapy. In brief, investigators identified target lesions representative of disease burden, calculated the sum of the longest diameter of all target lesions, and used this value as a baseline measure. The best overall response to therapy was then identified based on definitions outlined in the literature^3^; best overall responses comprised complete response (defined as the disappearance of all target lesions, with any pathological lymph nodes [target or nontarget] showing a reduction in the short axis to <10 mm), partial response (defined as ≥30% decrease in the sum of target lesion diameters, with the baseline sum used as the reference), progressive disease (defined as ≥20% increase in the sum of target lesion diameters, with the smallest sum on study used as the reference, plus an absolute ≥5-mm increase in the sum), and stable disease (defined as neither sufficient reduction to qualify for partial response nor sufficient increase to qualify for progressive disease, with the smallest sum of target lesion diameters used as the reference). Patients who had documented complete or partial response were classified as responders who experienced objective imaging response.

Survival analysis was performed based on patient response status. To avoid immortal time bias, only patients with a documented response were included in the survival analysis. Most imaging assessments were performed using contrast-enhanced computerized tomographic scanning at 12 weekly intervals, although physicians had final discretion on the timing and modality of imaging assessment.

Baseline demographic, histological, clinical, imaging, and laboratory data were captured using a standard template.^11^ We extracted IMDC risk factors (comprising hemoglobin level below the lower limit of normal, platelet count above the upper limit of normal, neutrophil count above the upper limit of normal, corrected calcium level above the upper limit of normal, Karnofsky Performance Status <80%, and time from diagnosis to initiation of systemic treatment <1 year), which are established prognostic factors associated with mRCC.^11^ The IMDC risk groups were assigned based on the number of risk factors present, with 0 factors indicating favorable risk, 1 or 2 factors indicating intermediate risk, and 3 or more factors indicating poor risk. Cytoreductive nephrectomy (CN) was defined as any nephrectomy performed before the receipt of systemic therapy, and deferred nephrectomy was defined as any nephrectomy performed after the receipt of systemic therapy. Data on educational level, race and ethnicity, and health insurance coverage were not collected because we had limited access to this information.

### Statistical Analysis

Associations between variables of interest and objective imaging response were assessed using χ^2^ tests, Fisher exact tests, and 1-way analysis of variance. The participant flowchart is available in eFigure 1 in the Supplement. Logistic regression analysis was used to simultaneously examine the associations between multiple potential baseline characteristics of interest and objective imaging response, along with the likelihood of objective imaging response based on the type of first-line IO combination therapy received. Time-to-event end points, such as OS and TTNT, were evaluated using the Kaplan-Meier method. Overall survival was calculated from the time of initiation of first-line therapy to death associated with any cause or the censored date of last follow-up. Time to next treatment was defined as the time from initiation of first-line therapy to initiation of second-line therapy or the censored date of last follow-up. The case-deletion method was used when missing data were encountered. All statistical tests were 2-sided with a significance threshold of *P* ≤ .05. Analyses were performed using SAS OnDemand for Academics, version 9.4 (SAS Institute Inc).

## Results

### Baseline Characteristics

The IMDC database consisted of 12 940 patients. Of those, 1085 patients received first-line IO combination therapies. Analyses of baseline characteristics, type of first-line IO combination therapy received, and their association with objective imaging response was performed using data from 899 patients (median age, 62.8 years [IQR, 55.9-69.2 years]; 666 of 898 male [74.2%], and 232 of 898 female [25.8%]) with evaluable responses. Survival analyses included 895 patients with evaluable responses and OS data, and the TTNT survival analysis included 894 patients with evaluable responses and TTNT data.

Baseline participant characteristics at the time of initiation of first-line therapy, grouped by objective response assessment, are shown in Table 1. A total of 381 participants had objective response to first-line therapy, and 518 participants did not. Among responders vs nonresponders, age (median, 62 years [IQR, 55-68 years] vs 63 years [IQR, 56-70 years]), sex (286 of 380 male [75.3%] vs 380 of 518 male [73.4%]), and the presence of sarcomatoid histological characteristics (66 of 309 participants [21.4%] vs 71 of 331 participants [21.5%]) were similar. Of 794 patients with data available on IMDC risk classification, 127 (16.0%) had favorable risk, 442 (55.7%) had intermediate risk, and 225 (28.3%) had poor risk. Compared with nonresponders, responders were more likely to have favorable IMDC risk (68 of 344 participants [19.8%] vs 59 of 450 participants [13.1%]; *P* = .001) and a higher prevalence of CN (89 of 378 participants [23.5%] vs 88 of 518 participants [17.0%]; *P* = .002) and lung metastases (289 of 379 participants [76.3%] vs 329 of 502 participants [65.5%]; *P* < .001).

**Table 1.** Baseline Participant Characteristics by Objective Imaging Response

Characteristic	Participants, No./total No. (%)		*P* value
	Nonresponders (n = 518)	Responders (n = 381)	
Age, median (IQR), y	63 (56-70)	62 (55-68)	.26
Sex			
Male	380/518 (73.4)	286/380 (75.3)	.56
Female	138/518 (26.6)	94/380 (24.7)	
Sarcomatoid histological characteristics	71/331 (21.5)	66/309 (21.4)	>.99
Clear cell histological characteristics	376/435 (86.4)	326/357 (91.3)	.03
First-line therapy			
IOIO	412/518 (79.5)	245/381 (64.3)	<.001
IOVE	106/518 (20.5)	136/381 (35.7)	
IMDC risk group			
Favorable	59/450 (13.1)	68/344 (19.8)	.001
Intermediate	244/450 (54.2)	198/344 (57.6)	
Poor	147/450 (32.7)	78/344 (22.7)	
IMDC risk factors			
KPS <80%	74/485 (15.3)	39/361 (10.8)	.06
Time from diagnosis to initiation of treatment <1 y	351/511 (68.7)	252/378 (66.7)	.52
Calcium level >ULN	67/458 (14.6)	41/342 (12.0)	.28
Hemoglobin level <LLN	272/486 (56.0)	173/368 (47.0)	.009
Platelet count >ULN	86/479 (18.0)	65/365 (17.8)	.96
Neutrophil count >ULN	77/473 (16.3)	35/357 (9.8)	.007
Baseline LDH >ULN	208/465 (44.7)	33/78 (42.3)	.55
Nephrectomy			
Cytoreductive	88/518 (17.0)	89/378 (23.5)	.002
Deferred	9/518 (1.7)	17/378 (4.5)	
Sites of metastasis			
>1 Site	377/469 (80.4)	296/347 (85.3)	.07
Lung	329/502 (65.5)	289/379 (76.3)	<.001
Lymph nodes	239/495 (48.3)	195/375 (52.0)	.28
Bone	181/497 (36.4)	114/377 (30.2)	.06
Liver	80/486 (16.5)	57/375 (15.2)	.62
Brain	33/487 (6.8)	16/376 (4.3)	.11
Adrenal	81/478 (16.9)	57/372 (15.3)	.52
Pancreas	50/477 (10.5)	31/371 (8.4)	.30
Spleen	3/470 (0.6)	3/368 (0.8)	.76
Comorbidities			
Preexisting autoimmune disease	6/216 (2.8)	6/212 (2.8)	>.99

Abbreviations: IMDC, International Metastatic Renal Cell Carcinoma Database Consortium; IOIO, immuno-oncology therapy with ipilimumab-nivolumab; IOVE, immuno-oncology therapy with immune checkpoint blockade plus vascular endothelial growth factor inhibitor combinations (including axitinib-avelumab, axitinib-pembrolizumab, cabozantinib-nivolumab, and lenvatinib-pembrolizumab therapies); KPS, Karnofsky Performance Status; LDH, lactate dehydrogenase; LLN, lower limit of normal; ULN, upper limit of normal.

With regard to best overall response among all participants, 37 (4.1%) had complete response, 344 (38.3%) had partial response, 315 (35.0%) had stable disease, and 203 (22.6%) had progressive disease. A total of 108 patients (12.0%) received treatment as part of a clinical trial; 657 patients (73.1%) received first-line IOIO therapy, and the remaining 242 patients (26.9%) received first-line IOVE therapy. Median follow-up from the time of first-line treatment initiation was 15.6 months (range, 1.2-88.0 months).

### Clinical Characteristics and Objective Response

**Figure 1.**
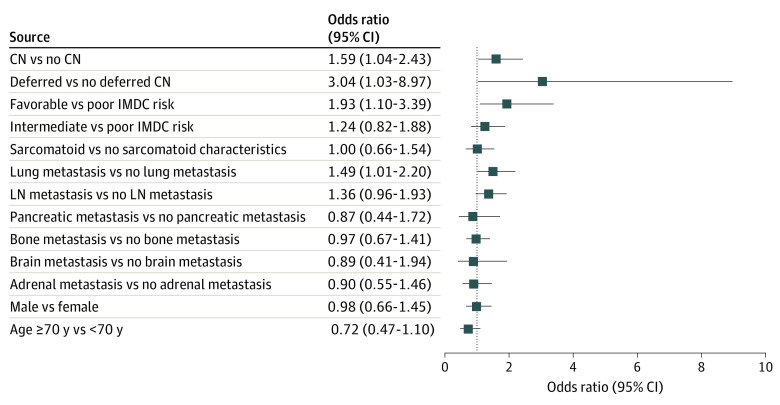
Association Between Baseline Characteristics and Objective Imaging Response Results from adjusted logistic regression analysis. Whiskers represent Wald 95% CIs. CN indicates cytoreductive nephrectomy; IMDC, International Metastatic Renal Cell Carcinoma Database Consortium; and LN, lymph node.

Baseline clinical characteristics of interest were defined a priori based on established associations with favorable OS or objective imaging response; these characteristics included IMDC risk group,^11^ presence of sarcomatoid histological characteristics,^12^ receipt of CN,^13^ and sites of metastasis.^14,15,16,17^ Although age^18,19^ and sex^20^ have not been reported to interact with outcomes associated with contemporary therapies for mRCC, these variables were included in the analysis because they had not been examined as confounders in large routine-practice data sets. In the adjusted logistic regression analysis, only CN (odds ratio [OR], 1.59; 95% CI, 1.04-2.43; *P* = .03), deferred nephrectomy (OR, 3.04; 95% CI, 1.03-8.97; *P* = .04), lung metastases (OR, 1.49; 95% CI, 1.01-2.20; *P* = .04), and favorable vs poor IMDC risk group (OR, 1.93; 95% CI, 1.10-3.39; *P* = .02) were associated with objective imaging response in a statistically significant and clinically meaningful manner (Figure 1).

**Figure 2.**
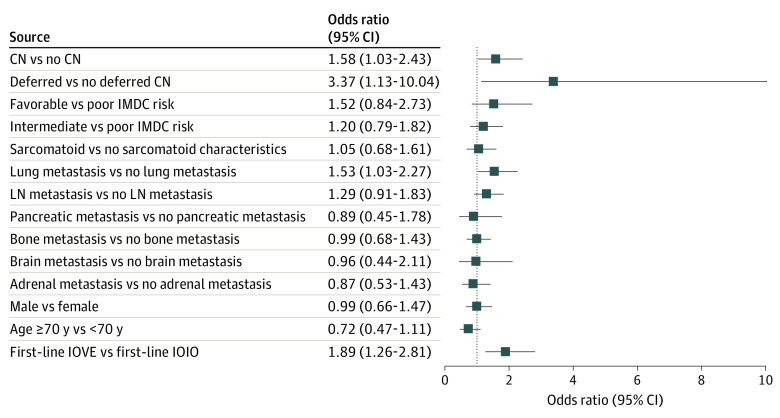
Association Between First-Line Immuno-oncology Combination Therapies and Objective Imaging Response Results from adjusted logistic regression analysis. Whiskers represent Wald 95% CIs. CN indicates cytoreductive nephrectomy; IMDC, International Metastatic Renal Cell Carcinoma Database Consortium; IOIO, immuno-oncology therapy with ipilimumab-nivolumab; IOVE, immuno-oncology therapy plus vascular endothelial growth factor inhibitor combinations (including axitinib-avelumab, axitinib-pembrolizumab, cabozantinib-nivolumab, and lenvatinib-pembrolizumab therapies); and LN, lymph node.

Treatment with IOVE vs IOIO (reference group) was independently associated with increased odds of obtaining objective imaging response (OR, 1.89; 95% CI, 1.26-2.81; *P* = .002) (Figure 2). When examining the overlapping indications for IOIO and IOVE therapies by assessing the 667 patients with intermediate and poor IMDC risk only, differences were identified in partial response (180 of 528 patients [34.1%] receiving IOIO vs 72 of 139 patients [51.8%] receiving IOVE; *P* < .001) and progressive disease (145 of 528 patients [27.5%] receiving IOIO vs 17 of 139 patients [12.2%] receiving IOVE; *P* < .001) (Table 2; eFigure 3 in the Supplement).

**Table 2.** Best Overall Response and Overall Survival by Type of First-Line Immuno-oncology Combination Therapy Among Patients With Intermediate and Poor International Metastatic Renal Cell Carcinoma Database Consortium Risk

Best overall response	Participants receiving IOIO, No. (%) (n = 528)	OS, median (95% CI), mo	1-y OS, %	Participants receiving IOVE, No. (%) (n = 139)	OS, median (95% CI), mo	1-y OS, %
Complete response	20 (3.8)	NE (32.9-NE)	100	4 (2.9)	NE (NE-NE)	100
Partial response	180 (34.1)	NE (29.2-NE)	93.3	72 (51.8)	44.1 (36.5-NE)	95.8
Stable disease	183 (34.7)	44.4 (35.1-NE)	90.7	46 (33.1)	31.6 (19.4-NE)	95.7
Progressive disease	145 (27.5)	8.4 (7.2-13.0)	50.3	17 (12.2)	18.5 (4.9-22.4)	64.7

Abbreviations: IOIO, immuno-oncology therapy with ipilimumab-nivolumab; IOVE, immuno-oncology therapy with immune checkpoint blockade plus vascular endothelial growth factor inhibitor combinations (including axitinib-avelumab, axitinib-pembrolizumab, cabozantinib-nivolumab, and lenvatinib-pembrolizumab therapies); OS, overall survival; NE, not estimable.

### Time-to-Event End Points and Objective Imaging Response

**Figure 3.**
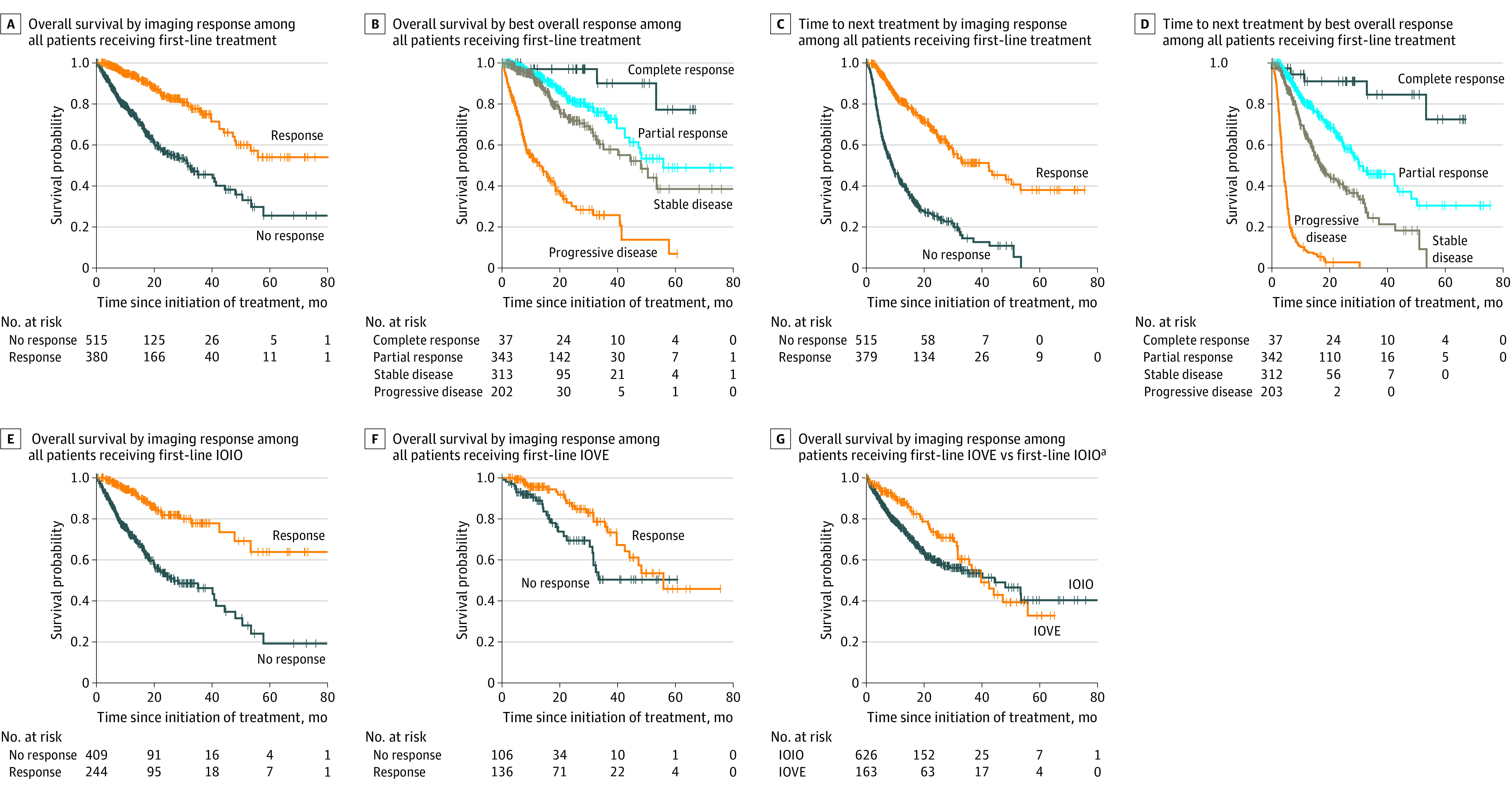
Kaplan-Meier Overall Survival and Time to Next Treatment Plus signs represent times of censoring. No imaging response includes stable or progressive disease, and imaging response includes complete or partial response. IOIO indicates immuno-oncology therapy with ipilimumab-nivolumab; and IOVE, immuno-oncology therapy plus vascular endothelial growth factor inhibitor combinations (including axitinib-avelumab, axitinib-pembrolizumab, cabozantinib-nivolumab, and lenvatinib-pembrolizumab). ^a^Only patients with intermediate and poor IMDC risk were included.

The association of objective imaging response with OS among all patients who received first-line treatment and experienced response vs nonresponse is shown in Figure 3A. Among responders vs nonresponders, median OS was not estimable (95% CI, 48.2 months to not estimable) vs 31.6 months (95% CI, 24.2-41.4 months; log rank *P* < .001). Overall survival based on best overall response is shown in Figure 3B. Median OS was not estimable (95% CI, 53.3 months to not estimable) among patients with complete response, 55.9 months (95% CI, 44.1 months to not estimable) among patients with partial response, 48.1 months (95% CI, 33.4 months to not estimable) among patients with stable disease, and 13.0 months (95% CI, 8.4-18.1 months) among patients with progressive disease (log rank *P* < .001). The overlapping OS curves for patients with vs without a documented response evaluation are shown in eFigure 2 in the Supplement. These survival curves confirmed that selection bias was not present because our findings were focused on patients with evaluable responses only.

The association of objective imaging response with prolonged TTNT is shown in Figure 3C. Responders had median TTNT of 42.4 months (95% CI, 30.0-53.4 months), whereas nonresponders had median TTNT of 8.9 months (95% CI, 7.9-11.0 months; log rank *P* < .001). Median TTNT also increased based on classification of best overall response; median TTNT was not estimable (95% CI, 53.5 months to not estimable) among patients with complete response, 30.1 months (95% CI, 27.4-43.5 months) among patients with partial response, 16.7 months (95% CI, 14.6-22.2 months) among patients with stable disease, and 3.9 months (95% CI, 3.4-4.3 months) among patients with progressive disease (log rank *P* < .001) (Figure 3D).

Overall survival curves for patients who received IOIO therapy with vs without objective imaging response are shown in Figure 3E. Objective imaging response was associated with improved OS, as observed in early and persistent separation of the survival curves; median OS was not estimable (95% CI, 53.4 months to not estimable) among responders vs 26.8 months (95% CI, 20.1-41.4 months) among nonresponders (log rank *P* < .001). A large proportion of patients in the IOIO group experienced progressive disease as the best overall response, with 145 patients (27.5%) having significantly reduced median OS of 8.4 months (95% CI, 7.2-13.0 months) (Table 2). In contrast, 17 patients (12.2%) in the IOVE group experienced progressive disease as the best overall response, with improved median OS of 18.5 months (95% CI, 4.9-22.4 months) (Table 2).

The OS curves comparing responders with nonresponders in the first-line IOVE group did not separate until 13 months, with median OS of 55.9 months (95% CI, 42.5 months to not estimable) among responders vs 33.1 months (95% CI, 25.4 months to not estimable) among nonresponders (log rank *P* = .02) (Figure 3F). This finding was associated with the low proportion of patients who experienced progressive disease (12.2%) and the favorable outcomes of patients with stable disease, who had median OS of 31.6 months (95% CI, 19.4 months to not estimable) and 1-year OS of 95.7%. Results of the OS analysis of patients in the IOIO vs IOVE groups restricted to their overlapping labeled indications (eg, intermediate and poor IMDC risk groups only) are shown in Figure 3G. Among patients with intermediate and poor IMDC risk, median OS was 44.4 months (95% CI, 32.9 months to not estimable) for those receiving IOIO therapy vs 42.5 months (95% CI, 31.6 months to not estimable) for those receiving IOVE therapy (log rank *P* = .15).

## Discussion

To our knowledge, this cohort study represents the largest analysis to date characterizing objective imaging response assessment in routine clinical practice and assess survival outcomes among patients with mRCC. Receipt of CN, presence of lung metastases, and favorable IMDC risk were independently associated with objective imaging response to contemporary first-line IO combination therapy approaches. Clinicians and patients can be informed that those with complete response had a median OS of not estimable, those with partial response had a median OS of 55.9 months, those with stable disease had a median OS of 48.1 months, and those with progressive disease had a median OS of 13.0 months. Time to next treatment was similarly prolonged among those with improved imaging response. Patients who experienced an objective response (ie, complete or partial response) had median OS that was not estimable compared with median OS of 31.6 months among patients who did not experience a response.

We compared the likelihood of objective imaging response between contemporary first-line IO combination therapies. Identification of a clinically meaningful increased OR of 1.89 (95% CI, 1.26-2.81; *P* = .002) if a patient received treatment with IOVE vs IOIO (reference) could have played a role in the early separation of the IOVE and IOIO survival curves (Figure 3G). Our findings were consistent with those of a recent network meta-analysis^21^ of clinical trial data that suggested patients receiving IOVE combination therapies had a higher likelihood of obtaining an imaging response compared with those receiving dual IOIO immunotherapy. The substantially worse median OS among patients who experienced progressive disease in our analysis (8.4 months among those receiving IOIO vs 18.5 months among those receiving IOVE) (Table 2) and the markedly lower percentage of patients with progressive disease who received IOVE (12.2%) vs IOIO (27.5%) therapies were also associated with this early separation in survival curves. However, it is notable that these survival curves eventually crossed, and no clear difference in OS was observed when examining the survival curves. This study did not conduct a formal statistical comparison of these 2 approaches because of the high number of substantial treatment selection biases that existed.

The association of objective imaging response with improvement in OS was maintained when IOIO and IOVE treatment approaches were examined separately (Figure 3E and F). As observed in the CheckMate 214 pivotal clinical trial,^8,22^ patients who did not respond to IOIO therapy died soon after beginning first-line treatment, with 50% of patients with progressive disease in the IOIO group dying within 1 year (Table 2). In contrast, patients who received IOVE combination therapies had an earlier initial plateau in their OS curves, regardless of response assessment, until 13 months, after which a significant separation in curves was noted. This finding suggests that VEGF inhibitors are able to stabilize disease in a clinically meaningful manner, producing benefit even without a documented imaging response.

The IMDC risk groups have been externally validated as predictors of survival benefit in the combination therapy era.^8,23,24^ To our knowledge, the present cohort study is the first to outline their association with survival benefit when examining objective imaging response across first-line combination therapy options. The clear separation of OS (Figure 3A) and TTNT (Figure 3C) curves based on response supports the importance of imaging response assessment in practice.

Cytoreductive nephrectomy was previously the standard of care in the cytokine therapy era.^25^ Postulated mechanisms of benefit included removal of a primary immunologic sink (in which potential tumor-specific circulating lymphocytes are diverted to the primary tumor and away from distant metastases)^26^ or reduction in neoplastic growth factors.^27^ The improved efficacy of systemic therapy with VEGF receptor blockade, outlined in the CARMENA (Cancer du Rein Metastatique Nephrectomie et Antiangiogéniques) clinical trial,^10^ challenged the role of CN in survival benefit. The CARMENA study established the noninferiority of systemic therapy with sunitinib vs CN followed by sunitinib. However, given insufficient accrual and high baseline incidence of poor-risk disease,^10^ the relevance of this work in the improved prognostic groups typically considered for CN is unclear. We identified a novel positive association between CN and objective imaging response. However, because of the biases associated with selection of candidates for CN (eg, IMDC risk and symptomatic burden),^28^ these selection characteristics may have confounded the association between CN and response. Given the known associations between tumor burden and response to ICB,^29,30^ achieving objective imaging response and improved OS may require ancillary approaches to reducing tumor bulk, such as CN. Caution is warranted when interpreting the identified association between deferred nephrectomy and objective imaging response because we found that this cohort of patients was highly selected in contemporary practice and required documentation of an objective imaging response before becoming eligible for nephrectomy.^31^

The clonal diversity of mRCC is broad, with distinct patterns of evolutionary biological characteristics associated with a wide range of survival outcomes. Predilection for metastases to liver and bone has been reported to confer worse prognosis in both genomically based prospective work^17^ and clinical evidence.^32^ The lung is the most common site of metastases in RCC,^33^ and the presence of lung metastases confers a survival advantage compared with other sites of disease.^14,17^ Cell lineage work profiling lung metastases has found dense lymphocytic infiltration into an immune-inflamed environment and an enhanced T-cell inflammatory signature with upregulated antigen presentation compared with alternative sites of metastases, such as bone and liver.^34^ Into this immune-rich environment, use of ICB-based first-line combination therapy strategies may therefore be able to estimate future imaging response.

### Strengths and Limitations

This study has strengths, including the use of a large data set from a 90% non–clinical trial population, which increases the immediate clinical relevance of these results. The low amount of missing data, depth of characterization of the underlying patient populations, and long follow-up period allow insight into typical imaging and survival outcomes that oncologists can use to help counsel and set treatment expectations.

The study also has limitations. These include a lack of independent blinded centralized imaging review, inconsistent intervals of imaging assessment, and an absence of imaging biomarkers. Routine-practice assessment of RECIST response may not adhere to the strict guidelines recommended for reporting of study results.

Although IMDC risk criteria and baseline characteristic–adjusted regression analysis were used to address some of the treatment selection biases associated with the comparison between IOIO and IOVE therapies, a number of confounders, such as imbalances in the burden of metastatic disease, were not accounted for and may have had implications for our results. Given that most IMDC sites are academic centers, our results may not be generalizable to the wider practicing oncology community. We had limited access to data on social factors associated with health, such as educational level, race and ethnicity, and private vs public health insurance coverage.

To avoid immortal time bias, only patients with evaluable responses were included in our analysis. Because the dates of imaging assessment were not standardized, imbalances may exist between cohorts of patients, producing measurement bias. If restaging intervals are heterogenous in our cohort, comparisons of conventional time-to-event end points, such as progression-free survival, will not be meaningful because the date of radiological evaluation for progression is a proxy for true progression time given that true progression occurs during the intervals between imaging assessments, resulting in interval-censored data.^35^ Later imaging assessment has been associated with prolonged progression-free survival.^36^ Given that the focus of our work was on absolute end points, such as OS, we hoped to minimize the consequences of such imbalances.

Our inability to confirm sarcomatoid histological characteristics, which are previously well-characterized factors associated with objective imaging response, may reflect the small sample and the incidence of sarcomatoid characteristics. Our favorable finding regarding the association between lung metastases and increased likelihood of objective imaging response may simply reflect the indolent biological processes associated with lung metastases.^37^

## Conclusions

In this cohort study nested in routine clinical practice, treating physician–assessed objective imaging response was associated with improved OS and TTNT among patients with mRCC. Treatment with IOVE therapy was associated with significantly higher odds of objective imaging response compared with IOIO therapy. The presence of lung metastases, receipt of CN, and favorable IMDC risk were associated with increased odds of objective imaging response. These findings may help inform treatment selection, especially in clinical contexts associated with high-volume multisite metastatic disease, in which obtaining objective imaging response is important.

## Notes

Supplement. **eFigure 1.** CONSORT Flow Diagram Outlining Participant Inclusion and Exclusion Steps at Each Stage of Data Analysis **eFigure 2.** Kaplan-Meier Overall Survival Curves for Patients With vs Without a Documented Imaging Response Evaluation **eFigure 3.** Adjusted Logistic Regression Analysis: Baseline Characteristics and Association With Imaging Response Including Intermediate vs Poor IMDC Risk Analysis
