# Supplementary material for: Imaging Response to Contemporary Immuno-oncology Combination Therapies in Patients With Metastatic Renal Cell Carcinoma
Source: JAMA Netw Open. 2022 Jun 10;5(6):e2216379. doi: 10.1001/jamanetworkopen.2022.16379 (PMC9187954; doi:10.1001/jamanetworkopen.2022.16379)
Supplement: Supplement. — eFigure 1. CONSORT Flow Diagram Outlining Participant Inclusion and Exclusion Steps at Each Stage of Data Analysis eFigure 2. Kaplan-Meier Overall Survival Curves for Patients With vs Without a Documented Imaging Response Evaluation eFigure 3. Adjusted Logistic Regression Analysis: Baseline Characteristics and Association With Imaging Response Including Intermediate vs Poor IMDC Risk Analysis [file jamanetwopen-e2216379-s001.pdf]

Supplementary Online Content

Navani V, Ernst M, Wells JC, et al. Imaging response to contemporary immuno-oncology combination therapies in patients with metastatic renal cell carcinoma. *JAMA Netw Open*. 2022;5(6):e2216379. doi:10.1001/jamanetworkopen.2022.16379

eFigure 1. CONSORT Flow Diagram Outlining Participant Inclusion and Exclusion Steps at Each Stage of Data Analysis

eFigure 2. Kaplan-Meier Overall Survival Curves for Patients With vs Without a Documented Imaging Response Evaluation

eFigure 3. Adjusted Logistic Regression Analysis: Baseline Characteristics and Association With Imaging Response Including Intermediate vs Poor IMDC Risk Analysis

This supplementary material has been provided by the authors to give readers additional information about their work.

© 2022 Navani V et al. *JAMA Network Open*.

eFigure 1. CONSORT Flow Diagram Outlining Participant Inclusion and Exclusion Steps at Each Stage of Data Analysis

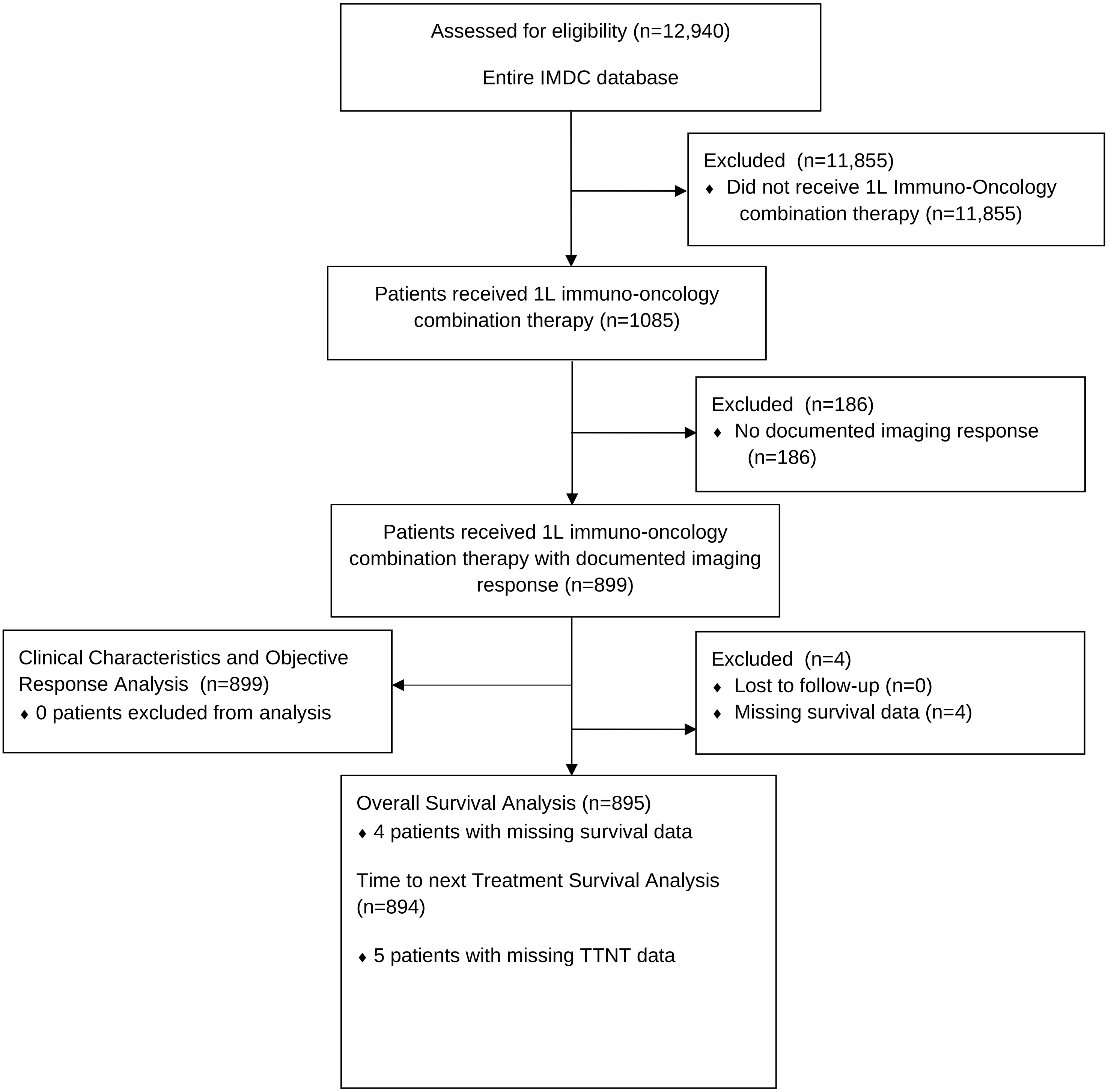

© 2022 Navani V et al. *JAMA Network Open*.

eFigure 2. Kaplan-Meier Overall Survival Curves for Patients With vs Without a Documented Imaging Response Evaluation

<u>Abbreviations Used:</u>
Survivalmon =Months since initiation of therapy; Docresponse 1 = Documented Imaging Response; Docresponse 0 = No Documented Imaging Response.

Monday, 11 April 2022 22:26:03 1

The LIFETEST Procedure

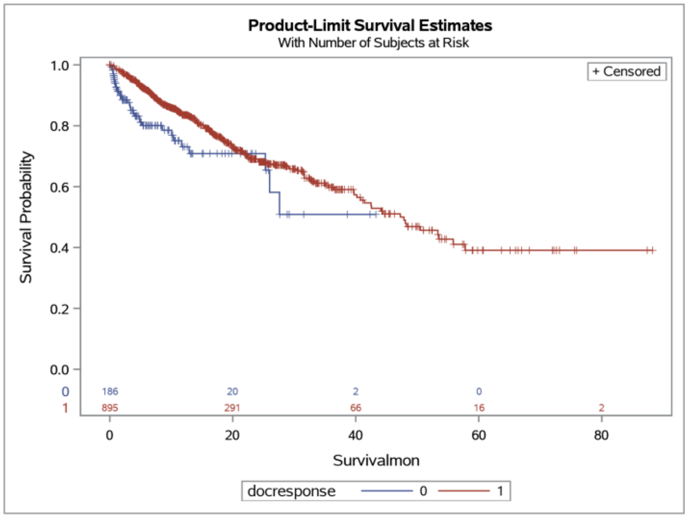

© 2022 Navani V et al. *JAMA Network Open*.

eFigure 3. Adjusted Logistic Regression Analysis: Baseline Characteristics and Association With Imaging Response Including Intermediate vs Poor IMDC Risk Analysis

Abbreviations Used:

IMDC – International Metastatic Renal Cell Carcinoma Database Consortium; CIs – Confidence Intervals; Cytoreductive 1 vs 0 = Cytoreductive Nephrectomy vs No Cytoreductive Nephrectomy; Cytoreductive 2 vs 0 = Deferred Nephrectomy vs No Deferred Nephrectomy; Heng 1 vs 2 = Favourable IMDC Risk vs Intermediate IMDC Risk; Heng 2 vs 3 = Intermediate IMDC Risk vs Poor IMDC Risk; Sarcomatoid = Sarcomatoid vs Non Sarcomatoid Histology; Lung Mets 1 vs 0 = Lung Metastases vs No lung Metastases; LymphNodesMet 1 vs 0 = Lymph Node Metastases vs No Lymph Node Metastases; PancMet 1 vs 0 = Pancreatic Metastases vs No Pancreatic Metastases; BoneMet 1 vs 0 = Bone Metastases vs No Bone Metastases; BrainMet 1 vs 0 = Brain Metastases vs No Brain Metastases; AdrenalMet 1 vs 0 = Adrenal Metastases vs No Adrenal Metastases; Male 1 vs 0 = Male vs Female; Old 1 vs 0 = Age >= 70 vs <70.

MVA Overall Population Monday, 11 April 2022 22:36:35 **4**

The LOGISTIC Procedure

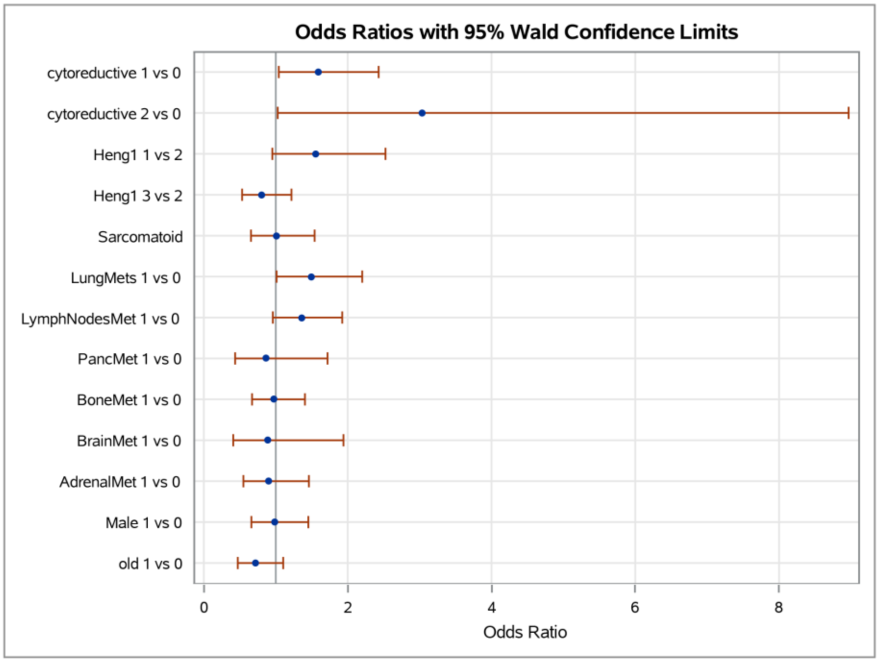

© 2022 Navani V et al. *JAMA Network Open*.
